# Venous thromboembolism in major trauma patients: a single‐center retrospective cohort study of the epidemiology and utility of D‐dimer for screening

**DOI:** 10.1002/ams2.290

**Published:** 2017-06-19

**Authors:** Tetsuya Yumoto, Hiromichi Naito, Yasuaki Yamakawa, Atsuyoshi Iida, Kohei Tsukahara, Atsunori Nakao

**Affiliations:** ^1^ Advanced Emergency and Critical Care Medical Center Okayama University Hospital Okayama Japan

**Keywords:** D‐dimer, deep venous thrombosis, pulmonary embolism, trauma, venous thromboembolism

## Abstract

**Aim:**

Venous thromboembolism (VTE) can be a life‐threatening complication after major trauma. The aim of this study was to investigate the epidemiology of VTE and to assess the usefulness of D‐dimer for screening for VTE in major trauma cases among the Japanese population.

**Methods:**

We examined a single‐center retrospective cohort of severely injured trauma patients who had been admitted to the emergency intensive care unit at Okayama University Hospital (Okayama, Japan) from April 2013 through to March 2016. Venous thromboembolism was confirmed by computed tomography angiography and computed tomography venography, which was determined based on the attending physician monitoring daily D‐dimer levels. Independent risk factors for VTE were determined by multiple logistic regression analysis. D‐dimer levels were evaluated using area under the receiver operating characteristic curve (AUROC) to predict VTE.

**Results:**

The study cohort consisted of 204 trauma patients (median Injury Severity Score, 20). Of the 204 patients, 65 (32%) developed VTE. The median time from admission to VTE diagnosis was 10 days. In multiple logistic regression analysis, higher Injury Severity Score and the presence of lower extremity fractures were revealed to be a risk factor for VTE. D‐dimer levels at day 10 showed moderate accuracy, of which the AUROC was 0.785 (95% confidence interval, 0.704–0.866; *P* < 0.001). The cut‐off that maximized the Youden index was 12.45 μg/mL.

**Conclusions:**

At least one of every three major trauma patients had potential development of VTE at a median of 10 days following admission to the intensive care unit. D‐dimer levels on day 10 can be a useful predictor of VTE.

## Introduction

Venous thromboembolism (VTE), which consists of deep venous thrombosis (DVT) and pulmonary embolism, is a common complication after major trauma associated with increased mortality and morbidity.^1^, ^2^ The incidence of VTE after trauma is reported to vary from 0.36% to 58%, which depends on patient demographics, study period, detection method, and use and type of VTE prophylaxis.^1^, ^3^, ^4^, ^5^, ^6^, ^7^ Incidence also significantly varies by race. White *et al*. and Zakai *et al*. showed that African‐Americans had a significantly higher incidence of VTE compared to Asian‐Pacific Islanders.^8^, ^9^ Wong *et al*.^10^ showed that the incidence of symptomatic VTE in the Asian trauma population was no less than that in Europe and North America. However, the epidemiology of VTE in major trauma cases among the Japanese population are poorly documented. In addition, although D‐dimer is useful in excluding DVT without the need for further diagnostic imaging, its predictability for VTE in major trauma has not been well established.^11^

We hypothesized that the incidence of VTE in a cohort of Japanese patients with major trauma would be as high as that of Europe and North America, and measuring daily D‐dimer levels would be applicable to those who developed VTE. Therefore, the aim of this study was to: (i) investigate the epidemiology of VTE, including identifying risk factors; (ii) define the cut‐off point of D‐dimer levels for screening for VTE in major trauma cases among the Japanese population.

## Methods

### Patients and study design

We undertook a single‐center retrospective cohort study of patients treated in the emergency intensive care unit (ICU) at Okayama University Hospital (Okayama, Japan). From April 2013 through to March 2016, consecutive trauma patients with an Injury Severity Score (ISS) greater than 8 were included.^1^ Patients who were younger than 16 years old or whose length of hospital stay was <3 days were excluded.^12^ Patients who required chronic dialysis, therapeutic anticoagulation for any reason, or limitations in therapy due to severe traumatic brain injury were also excluded. This study was approved by the Okayama University Hospital ethical committee (ID 1610‐508).

### Data collection

The following data or possible risk factors for VTE were recorded: age, sex, body mass index, mechanism of injury, daily D‐dimer levels, Abbreviated Injury Scale (AIS) for each of the six body regions, presence or absence of specific injuries (pelvic fracture, lower extremity fractures, and spinal cord injuries), need for massive transfusions, which was defined as transfusions of ≥10 units of red blood cells delivered within 24 h after hospital arrival, need for major surgery, ISS, central venous catheter placement days, mechanical ventilation days, length of ICU and hospital stay, and treatment of VTE.

### Definitions

Proximal DVT and distal DVT were defined as occurring above the knee and below the knee, respectively.^13^ Central venous catheter‐related thrombosis was defined according to whether a catheter had been present in the same or a contiguous venous segment within 72 h prior to the diagnosis.^14^ Major surgery was defined as one that lasted longer than 2 h.^5^

### Patient management

Intermittent pneumatic compression devices were applied to all patients after ICU admission unless complicated by leg injury. In the absence of major or relative contraindications, including active ongoing bleeding, coagulopathy, severe head injury, or non‐operatively managed solid organ injuries, chemical prophylaxis was initiated according to the decisions of the attending physician and operating team. Chemical prophylaxis was defined as i.v. or s.c. low molecular weight or unfractionated heparin, or s.c. selective Factor Xa inhibitor before any VTE diagnosis.^15^ Anticoagulant therapy for VTE was initiated after diagnosis with computed tomography (CT) unless the patient had a high risk of bleeding.

Screening with CT scan was fully based on the attending physician and team decision after considering the presence or absence of any symptoms of VTE or daily D‐dimer levels and trends, that is, swelling or tenderness in one leg, decreasing oxygen saturation, or the abrupt elevation or upward trend of D‐dimer levels after the third hospital day. However, we did not have specific criteria or a definitive guide to determine which patients underwent CT, in terms of D‐dimer levels.

Computed tomography imaging was carried out using an Aquilion 64‐row multislice CT (Toshiba Medical Systems, Okayama, Japan). A total of 100 mL contrast material was injected i.v. at a rate of 4 mL/s. Patients were scanned with 2‐mm‐thick sections from the external acoustic opening to the costophrenic angle during the arterial phase and early venous phase, which were 22 s and 120 s after the start of the injection of contrast material, respectively. Computed tomography venography was carried out with 3‐mm‐thick sections from the diaphragm to the feet at 300 s after the start of injection of contrast material. Presence of VTE was interpreted by a radiologist. D‐dimer levels were measured using a latex agglutination assay (April 2013–23 March, 2014: CS‐5100, Sysmex, Hyogo, Japan; 24 March, 2014–March 2016: ACL TOP, Mitsubishi Chemical Medience, Tokyo, Japan). Normal D‐dimer level using this assay was <1.0 μg/mL according to the manufacturer's instructions.

### Statistical analysis

Continuous variables are presented as median and interquartile range values, whereas categorical variables are shown as frequencies or percentages. Categorical variables were compared using Fisher's exact probability test. Mann–Whitney *U*‐test was used to evaluate variables with non‐normal distributions. For multiple comparisons with non‐normal distributions, Kruskal–Wallis followed by Bonferroni post‐test was used. Multiple logistic regression analysis was undertaken to identify risk factors for VTE adjusting covariates including age, sex, chest AIS of 4 or 5, ISS, presence or absence of spinal cord injuries or lower extremity fractures, and need for massive transfusions, which were chosen based on previous studies.^16^ The predictive ability of D‐dimer levels was estimated based on area under the receiver operating characteristic curve (AUROC). The suggested cut‐off value was determined using the Youden index. *P*‐values <0.05 were considered statistically significant. All analyses were carried out using IBM spss Statistics 22 (IBM SPSS, Chicago, IL, USA).

## Results

### Study cohort characteristics

**Figure 1:**
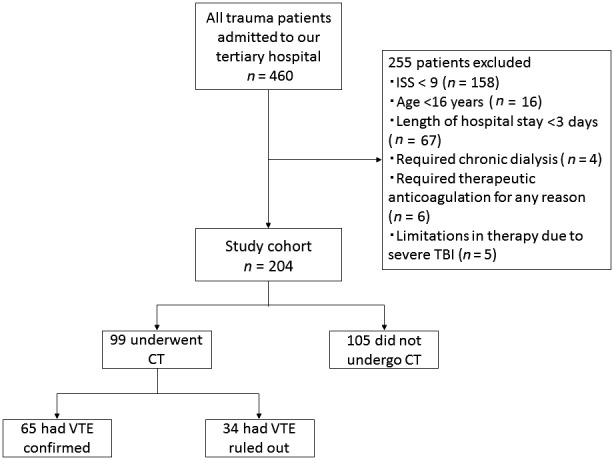
Flow diagram of study subjects, consisting of Japanese patients with major trauma, and workup for venous thromboembolism (VTE). CT, computed tomography; ISS, Injury Severity Score; TBI, traumatic brain injury.

During the 3‐year study period, a total of 460 trauma patients were admitted to the emergency ICU of our hospital. Of them, 204 patients met the inclusion criteria. Of the 204 patients, 99 (49%) were assessed for VTE using CT imaging due to elevated D‐dimer levels with likely clinical probability, of whom 65 patients were confirmed to have VTE. Figure 1 shows the derivation of the study population and workup for VTE. In the entire cohort, the incidence of VTE was 32% (65/204).

The demographics of the study group are summarized in Table 1. The patients with VTE was confirmed to be a severely injured cohort with significantly higher ISS scores and who required massive transfusions, major surgery, mechanical ventilation, and longer length of ICU and hospital stay. Patients with VTE showed significantly higher D‐dimer levels on days 1, 5, 7, 10, and 14.

**Table 1:** Demographics of study patients with major trauma in whom D‐dimer was retrospectively assessed for screening for venous thromboembolism (VTE)

Characteristic	All patients *n* = 204	(+) VTE *n* = 65	(−) VTE *n* = 139	*P*‐value
Age, years	49 (31, 67)	56 (40, 73)	43 (27, 67)	0.019
Male, *n* (%)	144 (71)	46 (71)	98 (71)	1.000
Body mass index, kg/m^2^	22.4 (20.3, 25.2)	22.8 (21.0, 25.7)	22.3 (20.2, 25.0)	0.371
Blunt mechanism of injury, *n* (%)	200 (98)	65 (100)	135 (97)	0.309
D‐dimer, μg/mL				
Day 1	19.5 (5.4, 45.7)	25.2 (10.9, 77.7)	13.4 (4.6, 40.5)	0.002
Day 5	6.4 (3.3, 11.3)	9.7 (5.5, 13.3)	5.2 (2.8, 8.9)	<0.001
Day 7	10.0 (5.8, 18.0)	13.6 (8.0, 22.5)	7.7 (3.9, 13.6)	<0.001
Day 10	13.0 (7.7, 24.9)	20.8 (13.2, 27.9)	9.3 (5.9, 13.5)	<0.001
Day 14	13.7 (8.7, 22.7)	19.5 (11.5, 29.2)	9.7 (7.0, 14.0)	<0.001
Head AIS of 4 or 5, *n* (%)	74 (36)	28 (43)	46 (33)	0.211
Chest AIS of 4 or 5, *n* (%)	51 (25)	26 (40)	25 (18)	0.002
Abdominal AIS of 4 or 5, *n* (%)	10 (5)	5 (8)	5 (4)	0.295
Pelvic fracture, *n* (%)	41 (20)	17 (26)	24 (17)	0.189
Lower extremity fracture, *n* (%)	47 (23)	22 (34)	25 (18)	0.020
Spinal cord injury, *n* (%)	12 (6)	6 (9)	6 (4)	0.203
Required MTs, *n* (%)	35 (17)	22 (34)	13 (9)	0.009
Major surgery, *n* (%)	127 (62)	49 (75)	78 (56)	0.009
Injury Severity Score	20 (13, 30)	30 (18, 38)	17 (13, 26)	<0.001
CVC placement, days	7 (2, 11)	7 (4, 11)	6 (0, 12)	<0.001
Length of ICU stay days	8 (4, 17)	15 (8, 25)	5 (3, 12)	<0.001
Length of hospital stay, days	18 (12, 28)	26 (19, 34)	15 (8, 22)	<0.001
Mechanical ventilation, *n* (%)	120 (59)	53 (82)	67 (48)	<0.001
Mechanically ventilated days	3 (0, 15)	13 (5, 25)	0 (0, 6)	<0.001
CT, *n* (%)	99 (49)	65 (100)	34 (24)	<0.001
VTE, *n* (%)	65 (32)			
PE, *n* (%)	33 (16)			
Proximal DVT, *n* (%)	44 (22)			
Distal DVT, *n* (%)	34 (17)			
CRT, *n* (%)	22 (34)			
Chemical prevention for VTE, *n* (%)	17 (8)	6 (9)	11 (8)	0.788

*P* values were calculated using Fisher's exact probability test or Mann–Whitney *U* test. Data are number (%) or median (interquartile range).

AIS, Abbreviated Injury Scale; CT, computed tomography; CVC, central venous catheter; CRT, central venous catheter‐related thrombosis; DVT, deep venous thrombosis; ICU, intensive care unit; MT, massive transfusion; PE, pulmonary embolism.

**Figure 2:**
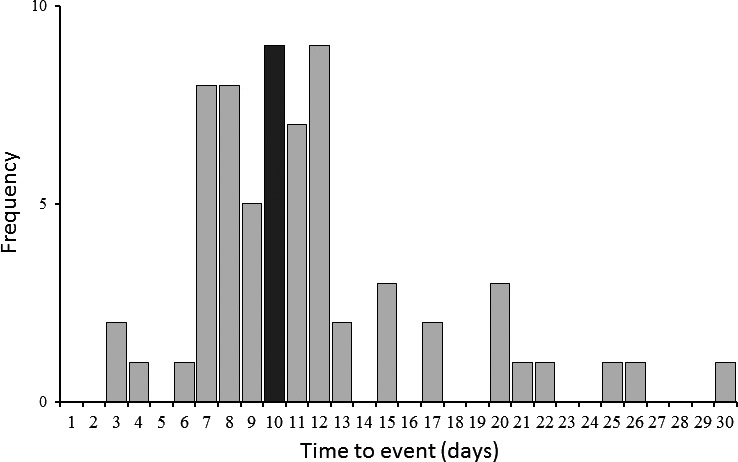
Time‐to‐event data for venous thromboembolism in Japanese patients with major trauma. Black bar indicates median value. The median time from admission to venous thromboembolism diagnosis was 10 days.

Time to event data for VTE is shown in Figure 2. The median time from admission to VTE diagnosis was 10 days. Venous thromboembolism was detected in 58 of the 65 patients at first CT examination and in 7 patients at the second or third exam. Of the 34 patients in whom VTE was not identified by CT image, 24 were examined by CT once and 10 were examined twice.

### Risk factors for VTE

Univariate analysis showed that risk factors for VTE included older age, lower extremity fractures, massive transfusions, chest AIS of 4 or 5, major surgery, higher ISS score, prolonged placement of central venous catheter, mechanical ventilation, and longer length of ICU or hospital stay (Table 1). In multiple logistic regression analysis, higher ISS and the presence of lower extremity fractures were revealed to be the significant risk factors for VTE (Table 2).

**Table 2:** Multiple logistic regression analysis of risk factors for venous thromboembolism in Japanese patients with major trauma

Risk factor	Odds ratio (95% CI)	*P*‐value
Age	1.02 (1.00–1.03)	0.078
Sex, male	1.33 (0.62–2.88)	0.464
Chest AIS of 4 or 5	1.24 (0.53–2.94)	0.622
Spinal cord injury	1.65 (0.43–6.29)	0.462
Lower extremity fracture	2.19 (1.02–4.70)	0.044
Injury Severity Score	1.06 (1.02–1.10)	0.004
Massive transfusion	2.38 (0.98–5.80)	0.055

AIS, Abbreviated Injury Scale; CI, confidence interval.

### Levels of D‐dimer for VTE screening

**Figure 3:**
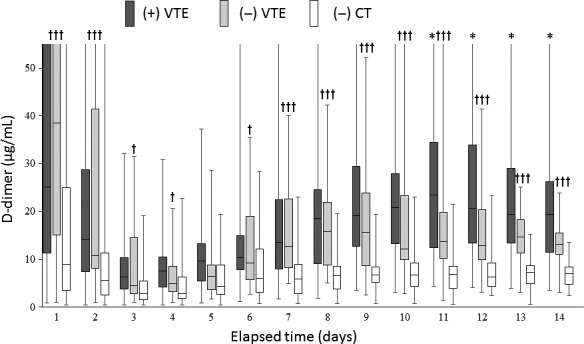
Changes in D‐dimer levels from time of injury to day 14 among Japanese patients with major trauma. Patients are grouped according to the presence of venous thromboembolism ((+) VTE), without VTE by computed tomography (CT) imaging ((−) VTE), and without CT examination ((−) CT). *P < 0.05, significant difference between (+) VTE and (−) VTE groups. †*P* < 0.05, †††*P* < 0.001, significant difference between (−) VTE and (−) CT image groups.

Time‐course changes of D‐dimer levels among patients with VTE, those without VTE by CT image, and those without CT examination are provided in Figure 3. From days 1 to 3, D‐dimer levels substantially decreased in all groups. Upward trend and higher D‐dimer levels were confirmed after day 3 among patients who were examined for VTE using CT image regardless of the presence or absence of VTE.

D‐dimer levels at day 10, which was the median time from admission to VTE diagnosis, showed moderate accuracy; the AUROC was 0.785 (95% confidence interval [CI]; 0.704–0.866; *P* < 0.001). The cut‐off that maximized the Youden index was 12.45 μg/mL with sensitivity and specificity of 0.723 (95% CI, 0.645–0.785) and 0.803 (95% CI, 0.720–0.869), respectively.

### Treatment and outcomes

Of the 65 patients with VTE, 58 (89%) who were considered to be at low risk of bleeding were treated with anticoagulants immediately after diagnosis of VTE. Forty‐two patients were treated with unfractionated heparin followed by warfarin (*n* = 9), and selective Factor Xa inhibitor (*n* = 6). All patients were asymptomatic and had no clinical manifestations at VTE diagnosis through hospital discharge. All patients in the cohort survived to hospital discharge.

## Discussion

This is the first study to investigate the epidemiology of VTE in major trauma cases in Japan. We found that VTE was detected in nearly one of every three major trauma patients at a median of 10 days following ICU admission. Higher ISS and the presence of lower extremity fractures were identified as the significant risk factors for VTE among a cohort of severely injured trauma patients. Our results also suggested that D‐dimer levels at 10 days following admission could be a useful predictor of VTE.

Venous thromboembolism potentially leads to life‐threatening complications after traumatic injury, which remains a major problem worldwide.^1^, ^2^ However, the epidemiology of VTE after major trauma in Japanese patients has not been well evaluated. Niikura *et al*.^17^ showed that the incidence of VTE in Japanese patients with pelvic and/or lower extremity fractures was 19%, whereas Masuda *et al*.^18^ showed that the incidence of DVT with cervical spinal cord injury was 10%. Our study found that the incidence of VTE among a cohort of trauma patients with severe injury (median ISS, 20) was 32%, which was as high as previously reported, or higher.^1^, ^2^, ^3^, ^4^, ^5^, ^6^, ^7^

Diagnosis and incidence of DVT depends on the method of detection. Either Doppler or duplex ultrasound screening have been used as the gold standard in major studies.^11^, ^12^, ^14^ Although ultrasound can be carried out at the bedside non‐invasively without contrast material, the diagnostic accuracy is extremely sensitive to the experience of the examiner.^19^ Also, associated injuries often limit the complete screening, which possibly leads to poor accuracy in the detection of DVT.^20^ Combined CT pulmonary angiography and CT venography, as used in our study, have advantages over ultrasound in terms of one test for both DVT and pulmonary embolism and high diagnostic accuracy.

As CT is not always available, however, D‐dimer is often used for VTE screening.^21^ Niikura *et al*.^22^ showed the usefulness of D‐dimer in predicting VTE in patients with pelvic or lower extremity fractures that require surgery. The cut‐off level of D‐dimer was 15.2 μg/mL at day 7 after injury. Our results suggested that the measurement of D‐dimer at approximately 10 days after trauma could be helpful in determining further diagnostic research to identify VTE.

Various risk factors have been identified as being associated with VTE in trauma patients.^1^, ^5^, ^23^, ^24^, ^25^ Our results, in which higher ISS and the presence of lower extremity fractures were the significant risk factors for VTE, were consistent with data from prior studies.^16^

This study has several limitations. First, there were no defined criteria in terms of D‐dimer levels to perform CT imaging, which was considered to be a weak point of this retrospective study. Although the patient group that did not undergo CT, who were considered unlikely to have VTE, showed significantly lower D‐dimer levels over the clinical course compared with the CT group, it might be insufficient to exclude the diagnosis of VTE. Heterogeneity of traumatic injuries would prevent CT examination for all patients at a proper time with strict protocol adherence. Second, we measured only short‐term outcomes. Although the rate of VTE in patients with pelvic fractures, vertebral fractures, or spinal cord injuries was highest in the first 3 months after injury,^26^ the risk of VTE persists for up to 1 year in patients with traumatic brain injury.^27^ Some patients in our study might be discharged prior to development of VTE. Third, introduction of thromboprophylaxis and initiation of anticoagulants for VTE were completely at the attending physician's discretion and operating team; therefore, we could not suggest any optimal management for VTE from our study. Finally, consideration of the cost‐effectiveness of VTE management should be required.

Nevertheless, we were the first to investigate the epidemiology of VTE and confirm the clinical effectiveness of D‐dimer for screening for VTE in major trauma cases among Japanese patients. The incidence of VTE in major trauma was as high as previous reports, or higher.

Assessment of this life‐threatening complication after major trauma using D‐dimer assay may be helpful for risk management in the rehabilitation setting. Future studies are required to identify further effective VTE screening and management strategies.

## Conclusions

Our findings suggested that VTE was assumed to be potentially detected in nearly one of every three major trauma patients at a median of 10 days following ICU admission. Higher ISS and the presence of lower extremity fractures were determined as significant risk factors for VTE among a cohort of severely injured trauma patients. Our results also suggested that D‐dimer levels at 10 days following admission could be a useful predictor of VTE.

## Disclosure

Conflict of Interest: None declared.

## References

[1] Geerts WH , Code KI , Jay RM , Chen E , Szalai JP . A prospective study of venous thromboembolism after major trauma. N. Engl. J. Med. 1994; 331: 1601–6. 7969340 10.1056/NEJM199412153312401

[2] Paffrath T , Wafaisade A , Lefering R *et al* Venous thromboembolism after severe trauma: incidence, risk factors and outcome. Injury 2010; 41: 97–101. 19608183 10.1016/j.injury.2009.06.010

[3] Shorr AF , Ramage AS . Enoxaparin for thromboprophylaxis after major trauma: potential cost implications. Crit. Care Med. 2001; 29: 1959–65. 10.1097/00003246-200109000-00001 11546959

[4] Schultz DJ , Brasel KJ , Washington L *et al* Incidence of asymptomatic pulmonary embolism in moderately to severely injured trauma patients. J. Trauma 2004; 56: 727–31. 15187734 10.1097/01.ta.0000119687.23542.ec

[5] Knudson MM , Ikossi DG , Khaw L , Morabito D , Speetzen LS . Thromboembolism after trauma: an analysis of 1602 episodes from the American College of Surgeons National Trauma Data Bank. Ann. Surg. 2004; 240: 490–6. 15319720 10.1097/01.sla.0000137138.40116.6c PMC1356439

[6] Bush S , LeClaire A , Hampp C , Lottenberg L . Review of a large clinical series: once‐ versus twice‐daily enoxaparin for venous thromboembolism prophylaxis in high‐risk trauma patients. J. Intensive Care Med. 2011; 26: 111–5. 21257630 10.1177/0885066610384462

[7] Berndtson AE , Costantini TW , Smith AM , Kobayashi L , Coimbra R . Does sex matter? Effects on venous thromboembolism risk in screened trauma patients. J. Trauma Acute Care Surg. 2016; 81: 493–9. 27280942 10.1097/TA.0000000000001157

[8] White RH , Keenan CR . Effects of race and ethnicity on the incidence of venous thromboembolism. Thromb. Res. 2009; 123(Suppl 4): S11–7. 19303496 10.1016/S0049-3848(09)70136-7

[9] Zakai NA , McClure LA . Racial differences in venous thromboembolism. J. Thromb. Haemost. 2011; 9: 1877–82. 21797965 10.1111/j.1538-7836.2011.04443.x

[10] Wong TH , Koh MP , Ng J . Symptomatic venous thromboembolism in Asian major trauma patients: incidence, presentation and risk factors. Eur. J. Trauma Emerg. Surg. 2013; 39: 495–500. 26815446 10.1007/s00068-013-0292-4

[11] Wells PS , Anderson DR , Rodger M *et al* Evaluation of D‐dimer in the diagnosis of suspected deep‐vein thrombosis. N. Engl. J. Med. 2003; 349: 1227–35. 14507948 10.1056/NEJMoa023153

[12] Adams RC , Hamrick M , Berenguer C , Senkowski C , Ochsner MG . Four years of an aggressive prophylaxis and screening protocol for venous thromboembolism in a large trauma population. J. Trauma 2008; 65: 300–6. 18695464 10.1097/TA.0b013e31817cf744

[13] Johnson SA , Stevens SM , Woller SC *et al* Risk of deep vein thrombosis following a single negative whole‐leg compression ultrasound: a systematic review and meta‐analysis. JAMA 2010; 303: 438–45. 20124539 10.1001/jama.2010.43

[14] PROTECT Investigators for the Canadian Critical Care Trials Group and the Australian and New Zealand Intensive Care Society Clinical Trials Group , Cook D , Meade M , Guyatt G *et al* Dalteparin versus unfractionated heparin in critically ill patients. N. Engl. J. Med. 2011; 364: 1305–14. 21417952 10.1056/NEJMoa1014475

[15] Scudday T , Brasel K , Webb T *et al* Safety and efficacy of prophylactic anticoagulation in patients with traumatic brain injury. J. Am. Coll. Surg. 2011; 213: 148–54. 21459632 10.1016/j.jamcollsurg.2011.02.027

[16] Rogers FB , Cipolle MD , Velmahos G , Rozycki G , Luchette FA . Practice management guidelines for the prevention of venous thromboembolism in trauma patients: the EAST practice management guidelines work group. J. Trauma 2002; 53: 142–64. 12131409 10.1097/00005373-200207000-00032

[17] Niikura T , Lee SY , Oe K *et al* Venous thromboembolism in Japanese patients with fractures of the pelvis and/or lower extremities using physical prophylaxis alone. J. Orthop. Surg. (Hong Kong) 2012; 20: 196–200. 22933678 10.1177/230949901202000212

[18] Masuda M , Ueta T , Shiba K , Iwamoto Y . D‐dimer screening for deep venous thrombosis in traumatic cervical spinal injuries. Spine J. 2015; 15: 2338–44. 26130085 10.1016/j.spinee.2015.06.060

[19] Shiver SA , Lyon M , Blaivas M , Adhikari S . Prospective comparison of emergency physician‐performed venous ultrasound and CT venography for deep venous thrombosis. Am. J. Emerg. Med. 2010; 28: 354–8. 20223396 10.1016/j.ajem.2009.01.009

[20] Davidson BL , Elliott CG , Lensing AW . Low accuracy of color Doppler ultrasound in the detection of proximal leg vein thrombosis in asymptomatic high‐risk patients. The RD Heparin Arthroplasty Group. Ann. Intern. Med. 1992; 117: 735–8. 1416575 10.7326/0003-4819-117-9-735

[21] Owings JT , Gosselin RC , Battistella FD , Anderson JT , Petrich M , Larkin EC . Whole blood D‐dimer assay: an effective noninvasive method to rule out pulmonary embolism. J. Trauma 2000; 48: 795–800. 10823521 10.1097/00005373-200005000-00001

[22] Niikura T , Sakai Y , Lee SY *et al* D‐dimer levels to screen for venous thromboembolism in patients with fractures caused by high‐energy injuries. J. Orthop. Sci. 2015; 20: 682–8. 25797331 10.1007/s00776-015-0711-y

[23] Buerger PM , Peoples JB , Lemmon GW , McCarthy MC . Risk of pulmonary emboli in patients with pelvic fractures. Am. Surg. 1993; 59: 505–8. 8338280

[24] Hill SL , Berry RE , Ruiz AJ . Deep venous thrombosis in the trauma patient. Am. Surg. 1994; 60: 405–8. 8198328

[25] Anderson FA Jr , Spencer FA . Risk factors for venous thromboembolism. Circulation 2003; 107: I9–16. 12814980 10.1161/01.CIR.0000078469.07362.E6

[26] Godat LN , Kobayashi L , Chang DC , Coimbra R . Can we ever stop worrying about venous thromboembolism after trauma? J. Trauma Acute Care Surg. 2015; 78: 475–81. 25710416 10.1097/TA.0000000000000556

[27] Olufajo OA , Yorkgitis BK , Cooper Z *et al* How long should we fear? Long‐term risk of venous thromboembolism in patients with traumatic brain injury. J. Trauma Acute Care Surg. 2016; 81: 71–8. 27015575 10.1097/TA.0000000000001046

